# Designing Electric Field Responsive Ultrafiltration Membranes by Controlled Grafting of Poly (Ionic Liquid) Brush

**DOI:** 10.3390/ijerph17010271

**Published:** 2019-12-30

**Authors:** Tejas Tripathi, Mohanad Kamaz, S. Ranil Wickramasinghe, Arijit Sengupta

**Affiliations:** 1 Ralph E Martin Department of Chemical Engineering, University of Arkansas, Fayetteville, AR 72703, USA; tejas.tripathi6@gmail.com (T.T.); makamaz@email.uark.edu (M.K.); swickram@uark.edu (S.R.W.); 2 Radiochemistry Division, Bhabha Atomic Research Centre, Mumbai 400085, India; 3 Chemical Sciences, Homi Bhabha National Institute, Mumbai 400094, India

**Keywords:** local perturbation, localized heating, electric responsive membrane, poly (ionic liquid)

## Abstract

Electric responsive membranes have been prepared by controlled surface grafting of poly (ionic liquid) (PIL) on the commercially available regenerated cellulose ultrafiltration membrane. The incorporation of imidazolium ring on membrane surface was evidenced by FTIR (Fourier transformed infra-red) and EDX (energy-dispersive X-ray) spectroscopy. The PIL grafting resultedin a rougher surface, reduction in pore size, and enhancement in hydrophilicity. The interaction of the electric field between the charged PIL brush and the oscillating external electric field leads to micromixing, and hence it is proposed to break the concentration polarization. This micromixing improves the antifouling properties of the responsive membranes. The local perturbation was found to decrease the water flux, while it enhanced protein rejection. At a higher frequency (1kHz) of the applied electric field, the localized heating predominates compared to micromixing. In the case of a lower frequency of the applied electric field, more perturbation can lead to less permeability, whereas it will have a better effect in breaking the concentration polarization. However, during localized heating at a higher frequency, though perturbation is less, a heating induced reduction in permeability was observed. The electric field response of the membrane was found to be reversible in nature, and hence has no memory effect.

## 1. Introduction

Water treatment, being a global challenge, requires continuous efforts to develop new technologies. This is important not only to obtain sufficient amount of portable water, but also to manage large amount of aqueous waste generated from different industries. Membrane based separation process is one of the most competent and clean techniques in this area [1,2]. Membrane based separations are capable of removing suspended solids, proteins, and viruses from water. Ultrafiltration (UF) membranes are extensively used in biotechnological industries mostly for protein concentration [3]. Despite their numerous advantages, there are few limitations as well. During permeation through the membranes, several molecules attach to the membrane surface and pore walls leading to an obstructive layer that decreases the permeate flux. This phenomenon, referred to as fouling, is one of the major challenges faced by the membrane industry [4,5,6]. Several studies have been aimed at reducing fouling to decrease the adverse effects on membrane performance. The approaches are mostly focused on either altering the feed properties or chemically modifying the membrane surface. Surface modifications change the properties of the membrane surface, which is supposed to be in contact with the feed stream, and hence can reduce fouling by suppressing interactions between fouling agents in the feed and at the surface. Several membrane surface modification methods have been reported in this regard [7,8,9,10,11]. 

One notable class of surface modified membranes is stimuli responsive membranes [12,13,14]. These membranes alter their physiochemical characteristics with varying environment. Membranes have been designed to respond to the external stimuli, such as pH, temperature, and electric and magnetic fields. Stimuli responsive membranes have been extensively exploited for controlled drug-delivery. More recent studies have shown them to be advantageous in the water treatment domain, especially in terms of their improved fouling resistance. For water treatment application, magnetic responsive membranes prepared by grafting responsive nano-brushes were reported to improve membrane performance with better antifouling characteristics [15,16,17,18]. The movement of the magnetically responsive polymer chains results in mixing at the membrane surface, which disrupts concentration polarization and hence reduces the rate of cake formation. Unlike pH or temperature responsive membranes, magnetic and electric responsive ones do not depend on changes in the feed conditions to show response. Rather, they rely only on an external oscillating field. Stimuli responsive membranes in general can be prepared by a number of techniques. A common approach is by grafting polymer groups having functionalities, that can respond to the stimuli on the membrane surfaces. Various grafting techniques, such as ultraviolet assisted photochemical grafting and plasma-initiated grafting are commonly used [10,19]. 

Surface-initiated atom transfer radical polymerization (ATRP) is one such grafting technique, which can be particularly advantageous in development of stimuli responsive membranes. In ATRP, a radical initiator is affixed to the surface of the membrane [20,21,22]. The catalyst instigates a polymerization reaction by the reversible abstraction of an atom, and as the monomer is added from the surface, polymer chains begin to grow. The key advantage of ATRP is that, the polymer chain can easily be controlled in this process by varying initiation and polymerization times; eventually making it possible to control pore size of the membrane. Also, unlike degradable surface modifications, the covalent grafting in ATRP is permanent.

Ionic liquid is considered to be the ‘green’ alternative of the volatile organic compounds due to the favorable properties like low vapour pressure, wide liquidus range, high degree of chemical, electrochemical, and thermal stability, impressive power of solubilizing organic, inorganic, and even some polymeric matrices [23,24,25,26,27]. The application of functionalized ionic liquid for task specific applications has also been demonstrated to be highly effective [28,29]. The unique nature of ionic liquid also provides some unconventional chemistry, which was not otherwise reported for conventional chemicals. The high degree of tunability in ionic liquid by a slight modification in either the cationic or anionic part is another unique property, which makes it widely applicable in various field including organic synthesis, catalysis, separation and electrochemistry. The loss of either cationic or anionic part during ion exchange separation using ionic liquid is one of the major problems faced by researchers. The blending of unique properties of ionic liquid with the advantageous high durability of polymer has also been effective as poly (ionic liquid) (PIL) in membrane technology [30,31]. The additional antimicrobial properties of PILs provide significant resistance over biofouling and biofilm formation in membrane technology [32,33,34]. 

**Figure 1:**
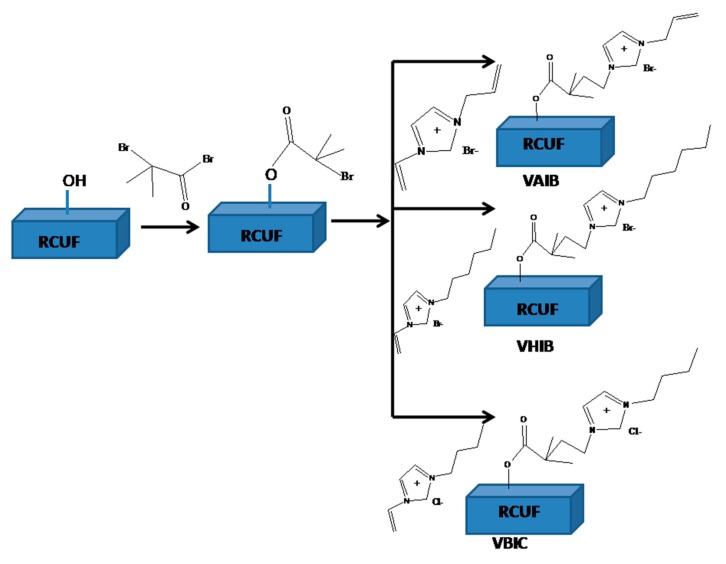
Schematic presentation of the modification for the preparation of electric responsive membranes. (RCUF: Regenerated Cellulose Ultrafiltration membrane; VAIB: vinyl allyl imidazolium bromide; VHIB: vinyl allyl imidazolium bromide and VBIC: vinyl butyl imidazolium chloride).

In this study, the controlled grafting of PILs was carried out on the surface of regenerated cellulose ultrafiltration (RC UF) membranes to obtain an electric response by the application of a remotely operated oscillating electric field. It is proposed that the electric interaction between these PIL brushes and the applied field would result in localized perturbation near the membrane boundary layer to avoid concentration polarization. Depending upon the frequency of the external electric field, localized heating can also be achieved to influence the membrane performance. Figure 1 schematically presents the proposed mechanism of the electric field responsive membranes.

## 2. Materials and Methods

Commercially available, composite membranes having a RC ultrafiltration layer on ultrahigh molecular weight polyethylene support and having a molecular weight cut-off of 300 kD were provided by the Millipore corporation. All membrane samples used in this study were cut into circular samples of 40 mm diameter from larger sheets. All membranes used were washed with deionized water (DI) prior to use.These chemicals were used as received from Sigma-Aldrich: Methanol (MeOH), Triethylamine (TEA), 4-dimethylaminopyridine (DMAP), copper (II) chloride (CuCl_2_, 99.999% trace metal basis), α bromoisobutyryl bromide (BIB, 98%), L-(+)-ascorbic acid (AA), 2,2’-bipyridine (BPy), Acetonitrile, Bovine serum albumin (BSA), 1-Vinylimidazole, allylbromide, hexyl bromide, and butyl chloride. Table 1 summarizes the dextran sample used and their molecular weights. The water used in all experiments was from a Milli-Q system. The ionic liquid monomers; 1-allyl-3-vinylimidazolium bromide, 1-hexyl-3-vinylimidazolium bromide, and 1 butyl-3-vinylimidazolium chloride henceforth will be termed as VAIB, VHIB, and VBIC, respectively.

### 2.1. Preparation of Electric Responsive Membrane by Surface Initiated ATRP

RC membranes discs were washed thrice with methanol for 20 min to ensure that glycerine, which is used as a preservative in these membranes, is removed completely [35]. Then, the membranes were soaked in deionized water for 30 min to remove the methanol used before. These washed membrane discs were then rinsed 3 times with dry acetonitrile for 20 min. For initiator immobilization, 2-BIB was reacted with the hydroxyl groups of the RC membrane for 5 min and 10 min. The initiator immobilization time was varied as a parameter here. The conditions created were 100 mM 2-BIB, 100 mM triethylamine, and 5 mM 4-dimethylaminopyridine. After reaction, the initiator-functionalized membranes were washed 3 times in a 1:1 (v/v) MeOH/H_2_O mixture.

ATRP was performedto graft poly(VAIB), poly(VHIB), and poly(VBIC) chains from the surfaces of these initiator immobilized membranes. The reaction solution used in this step was consisted of respective monomer (0.5 M), CuCl_2_ (20 mM), BPy (50 mM) in a 1:1 (v/v) MeOH: H_2_O mixture. The synthesis of the ionic liquid monomers and their characterization have been discussed in detail in our previous publication [32]. A 3-neck round bottom flask was evacuated under vacuum and filled with argon gas thrice. One membrane disk was added in one go to the flask and under argon flush, then 25 mL of the reaction solution was added into it. 100 mM ascorbic acid solution in a 1:1 (v/v) MeOH/H_2_O mixture was prepared, of which 2 mL was added to the flask under argon flush. The flask was shaken vigorously for 20 s and then left to react for 6 hours. For quenching the reaction, the membrane was removed and moved to a washing solution of 1:1 (v/v) MeOH/H_2_O. The membranes were repeatedly washed with DI water before further use. The synthesized membranes have been denoted as Base, VAIB 5, VHIB 5, VBIC 5, VAIB 10, VHIB 10, VBIC 10, where 5 and 10 are referred to the initiator immobilization time, while the ATRP time was kept at 6 hours.

### 2.2. Characterization

The membranes were characterized using Fourier transformed infrared (FTIR) spectroscopy. FTIR was used to identify the changes in the functionality of the membrane surface caused due to modification. Data were obtained using an IR Affinity instrument (Shimadzu, Columbia, MD, USA) with a horizontal ZnSe accessory. FTIR spectra were averaged over 300 scans covering a range of 1000–4000 cm^−1^. Prior to analysis, the membranes were dried overnight in vacuum oven at 35 °C. Atomic force microscopic (AFM) technique was used to observe the membrane surface morphology. The root-mean-square (RMS) roughness values of unmodified and modified membranes were measured. Bruker, Santa Barbara, CA, equipped with Dimension Icon AF and NanoScope analysis program was used to analyze the AFM results. Scanning electron microscopic (SEM) image analyses were carried out using FEI Nova Nanolab 200 Duo-Beam Workstation (Hilsboro, OR, USA). A coating layer 10-nm of gold was employed on the membrane prior to SEM analysis. A 15-kV electron beam was used as a source. Water contact angles were measured using an OCA20 contact angle system (Dataphysics, Filderstadt, Germany) at room-temperature. The contact angle was measured using the sessile drop method: A 5μL water drop was lowered onto the membrane surface using a needle tip. Contact angles were calculated after 4 s using imaging software. The mean of measurements taken at 5 different points on the membrane surface was recorded. Energy-dispersive X-ray (EDX) microanalysis was used for the elemental analysis or chemical characterization of the membrane sample. EDX analysis was done using SPARCstation 5 apparatus (Sun Microsystems, Menlo Park, CA, USA). 

The surface charge was measured by zeta potential using dynamic light scattering (DLS): Zetasizer Nano ZS90, Malvern, UK. The instrument is equipped with a flat surface cell. All measurements were carried out in triplicate. The zeta potential was measure in a similar fashion as reported in our previous literatures [36,37,38]. In the first step, feed solution was prepared by mixing 20 mL of 10 mM standard NaCl solution with 200 μL of standard zeta potential measuring solution. This standard Zeta potential measuring solution was procured from instrument supplier. In the next step, the pH of the above solution was adjusted to feed pH on which zeta potential needs to be monitored. This pH adjustment was carried out by 0.1 M HCl or NaCl. Then, on a flat cell, which is a part of the sample introduction assembly, the membrane sample was clipped and put in an appropriate place, so that only the upper surface of the membrane would be in touch with the solution to measure the zeta potential of the upper surface. In case of asymmetric membrane, the active side should be in contact with the solution. Then, the full system was run for 10 min, so that no cell was completely filled with the solution, also ensuring the complete equilibrium condition. Then, the zeta potential was measured. In the present case, the zeta potential was measured at pH 7 only.

### 2.3. Permeate Flux Measurements

**Figure 2:**
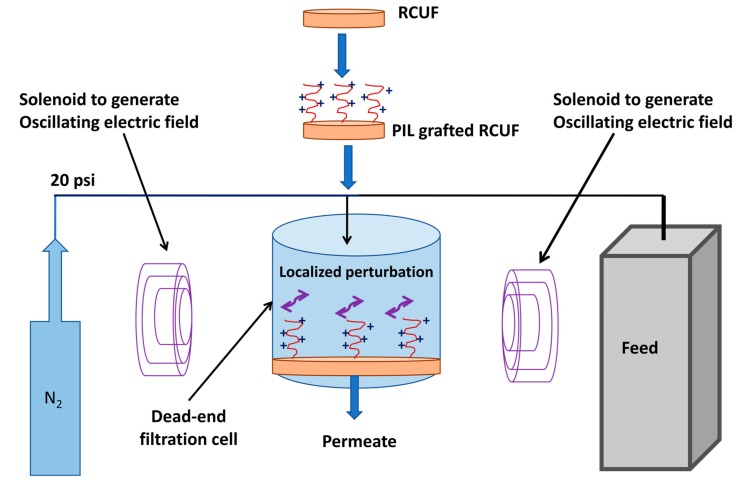
Schematic presentation of dead-end filtration set up for electric responsive membrane.

The permeate flux measurements with deionized (DI) water were carried out with the membranes using a 50 mL Amicon^TM^ ultrafiltration unit. Each membrane sample, after rinsing with DI water, was loaded into the cell, which was filled with feed solution and connected to a nitrogen tank using pressure tubes. Figure 2 schematically presents the filtration set up for electric responsive membrane in dead-end mode. The membrane was pre-compacted for 30 min at 40 psi (2.76 bar) pressure before being used for filtration flux measurement. Filtration was carried out at 20 psi (1.38 bar). The filtration cell was kept between two copper solenoids as shown in figure. These two solenoids were used to generate oscillating electric field. A software program (PLC, Click Koya, Automation Direct, Cumming, GA, USA) controlled the rate of power provided to the two solenoids. The frequency of current supplied to the solenoids determined the frequency of the alternating electric field. The solenoids were placed parallel and opposite to each other and perpendicular to the permeate flow. The permeate was collected in a beaker kept on a balance. The mass change during filtration was recorded in 1 min interval regularly up to 45 min. The permeate flux was calculated by monitoring the mass change (since the effective surface area for the membrane is known and the flux is volume/area/time. Since mostly we were using water or very dilute aqueous solution, therefore, the mass can be correlated with volume by taking into account the density term, which is 1 gm/cc for water. The electric field frequency was varied and, as proposed, there would be an electric field interaction between the positively charged PIL brushes and the oscillating applied electric field. This interaction would lead to localized perturbation near the membrane surface, as shown in the Figure 2. The permeation experiments were carried out first without any electric field, next in presence of 20 Hz oscillating electric field and then 1 kHz oscillating electric field and finally again without any electric field.

### 2.4. Rejection Studies

Aqueous solution of bovine serum albumin (BSA) having 1 g/L concentration was prepared as feed for rejection experiments. The permeate flux was measured according to the method as discussed in Section 2.3. Samples were collected for each filtration run at the end of 30 min. For the rejection study, an ultraviolet-visible (UV/Vis) spectrophotometer was used at a peak wavelength of 280 nm. A calibration curve was prepared for translating UV absorbance to BSA concentrations. The % rejection was calculated based on the equation reported in the literature [22]:

(1) $$\%\;R=\left(1-\;\frac{C_{p}}{C_{f}}\;\right)\times \;100\;\%$$

where, *C_p_* is the BSA concentration in the permeate and *C_f_* is the concentration of Bovine serum albumin (BSA) in the feed. The filtration experiments were also carried out with dextran solutions. In order to determine rejection curves, solutions of varying molecular weight were prepared. The fractions used are listed in Table 1 along with their molecular weights and concentrations. The samples were analyzed by high performance liquid chromatography (HPLC) to determine dextran concentrations before and after permeation. Size exclusion chromatography was employed in order to achieve sieving curve to find out the pore size. The instrument used was High Performance Liquid Chromatography (HPLC) instrument of 1260 Infinity procured from Agilent Technologies, Santa, Clara, CA, USA. The Shodex (SB-806 HQ) column was employed for present investigation, whereas 0.5 M sodium phosphate monobasic solution was employed as eluting solution with a flow rate of 0.8 mL/min and the column temperature 40 °C. High purity dextran of different molecular weights ranging from 6 kDa to 500 kDa in the phosphate buffer was used for establishing the calibration curves. 

## 3. Results and Discussion

### 3.1. Synthesis of Electric Responsive Membrane

The RC UF membranes have surface –OH groups, which have been utilized in the subsequent modification. The acid bromide group of the initiator (BIB) was attacked by the lone pair of electrons on the –OH group residing on the surface of the RC UF membrane, which subsequently led to ester moieties. The polymer brush grew at the location, where the initiator gets immobilized. The duration of the initiator immobilization significantly influenced the density of the PIL brush on the membrane surface [39]. In the next step, the activator generated atom transfer radical polymerization was utilized to graft PIL brush. This process is based on the slow equilibration of Cu ions in two different oxidation states [40]. The AGET (activator generated electron transfer) modification utilizes ascorbic acid as a reducing agent to resolve the issue of the stringent requirement of an oxygen/oxidant free environment. The vinylic double bond associated with each ionic liquid monomer was utilized for propagation of PIL chain. It is required to optimize the chain density as well as chain length in order to have optimum localized perturbation to break concentration polarization. 

### 3.2. Membrane Surface Characterization

**Figure 3:**
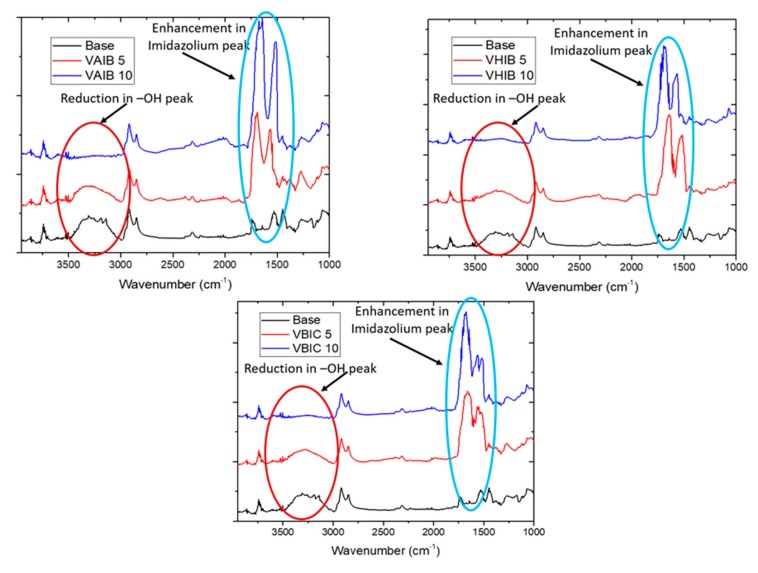
FTIR Spectra for base and modified membranes.

Figure 3 shows the FTIR spectra for the electric responsive membranes. The broad peak around 3300–3500 cm^−1^ for base membrane was attributed to the –OH groups of regenerated cellulose. On modification, this peak was found reduce considerably as the –OH group was utilized in initiator immobilization and used as site for the subsequent growth of PIL brush in ATRP step. The peak with shoulder near 1600 cm^−1^ was attributed to the imidazolium stretching, as reported in the literature [32]. With increase in ATRP time, the length of PIL brush increases and as a result, the peak intensity also increases. 

**Figure 4:**
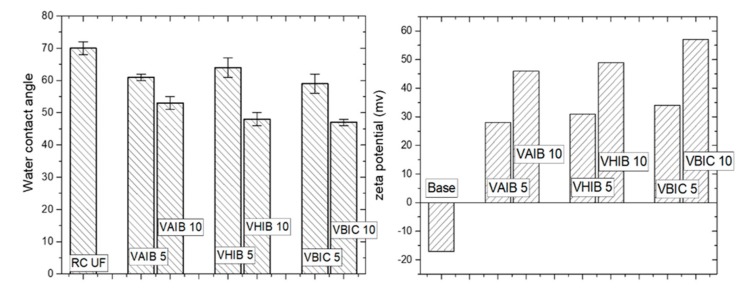
Water contact angles and the zeta potential for the responsive membranes.

The hydrophilicity/hydrophobicity of the membrane surface have been investigated by the measurement of water contact angle in static mode. The base RC-UF membrane was shown to have water contact angle of 70°, indicating the hydrophilic nature of the membrane, which is attributed to the presence of hydroxyl groups. However, the water contact angle further reduces by grafting the PIL brush on the membrane surface. The repeated unit of ionic moieties induce the electrostatic interaction between the PIL brush and the water molecules, hence improving the hydrophilicity of the resulting responsive membranes. The membrane surface with denser polymer chain implies more number of repeated ionic imidazolium moieties and hence the surface hydrophilicity further increases. Not much difference in the water contact angle was observed for different PIL brush. Figure 4 is showing the water contact angles for different responsive membranes. The zeta potential on the membrane surface also corroborated the same observation. The base membrane was found to have a negative zeta potential, which indicates the presence of hydroxyl groups (–OH). However, the zeta potential was found to be positive for all the responsive membranes. The membranes with a longer PIL brush were found to have a more positive zeta potential. All the zeta potential measurements were carried out at a feed of pH 7. Though the PIL brush has the positively charged imidazolium moiety and halide as counter ion, the electrostatic interaction between the imidazolium ion and the halide ion is very weak in nature. In presence of water, this mobile counter anion gets detached from the PIL brush. Hence, the positively charged imidazolium ions were exposed from the surface. This makes the membrane surface highly positively charged. The membrane surface with a denser PIL chain demonstrated a higher number of such imidazolium cationic moieties and hence the surface positive charge was found to enhance.

**Figure 5:**
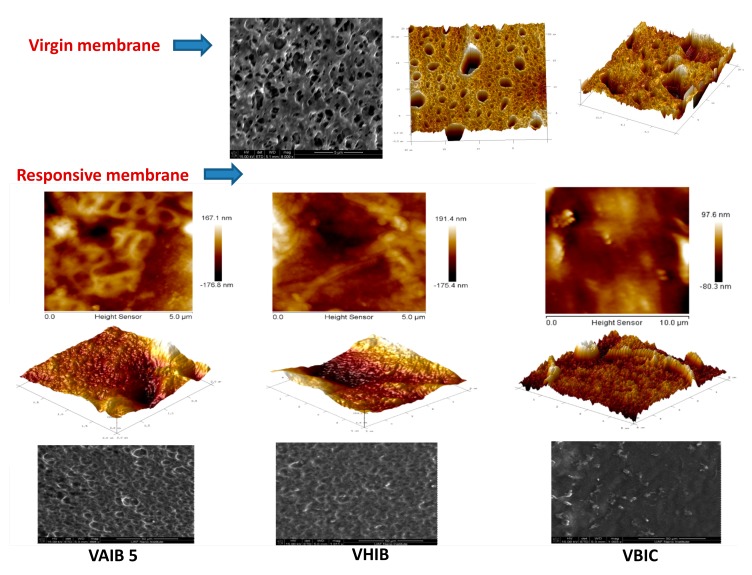
AFM and SEM images of virgin and responsive membranes.

To understand the surface morphology of the resulting responsive membranes, an atomic force microscopic technique (2D and 3D of membrane surface) and scanning electron microscopic technique were employed (Figure 5). The AFM and SEM of responsive membranes were compared with that of virgin membrane. In virgin membrane, the surface was found to be more porous with a distribution of pore size. The surface roughness was found to be less than those of responsive membranes. An appreciable change in surface morphology was noticed between virgin and modified membranes. Table 2 is summarizing the results of EDX analysis. 

The grafting of the PIL brush was found to reduce the overall pore size of the base membrane, generating a rougher surface. The ridge valley structure was observed for the responsive membrane surface. Surface roughness is a component of surface texture, measured in terms of deviations in the direction of the normal vector of a real surface from its ideal form. If these deviations are large, the surface is rough, and if they are small, the surface is smooth. The surface roughness calculated in the present case is actually the root mean squared roughness.

(2) $$R_{rms}=\;\sqrt{\frac{1}{n}\;\sum_{i=1}^{n}y_{i}^{2}}$$

where, *n* is the ordered equally spaced *y_i_* is the vertical distance from the mean line to the *i*th data point. This was calculated by scanning the membrane surface using AFM. The instrument associated software evaluated the root mean squared surface roughness. The roughness of the base RC UF membrane was evaluated as 16.4 nm, which was appreciably enhanced for the responsive membranes obtained by 5 min ATRP (VAIB 5 = 18.1 nm, VHIB 5 = 18.6 nm and VBIC 5 = 19.2 nm). The responsive membranes with higher grafting density (longer initiator immobilization time) resulted even more rough surface (VAIB 10 = 21.5 nm, VHIB 10 = 23.7 nm and VBIC = 26.1 nm).

### 3.3. Membrane Performance

**Figure 6:**
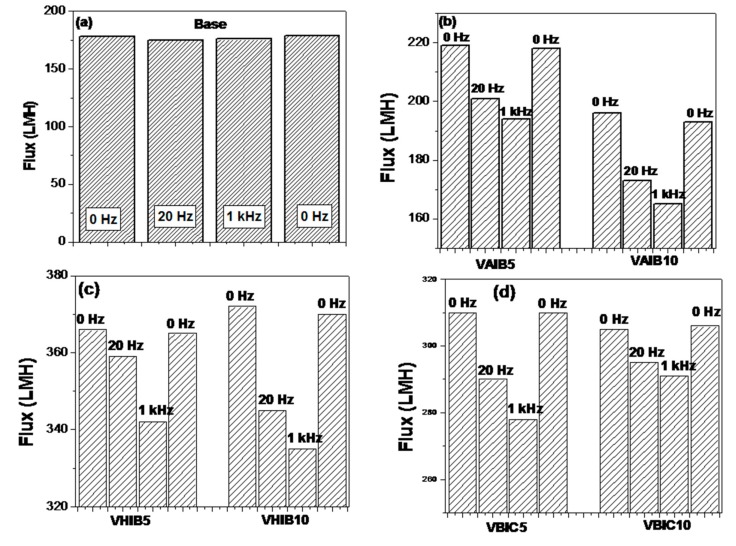
Water flux as a function of time for responsive membrane at different oscillating electric field frequency. (**a**): Virgin RCUF membrane; (**b**): Responsive membranes originated from vinyl allyl imidazolium bromide; (**c**): Responsive membranes originated from vinyl hexyl imidazolium bromide; (**d**): Responsive membranes originated from vinyl butyl imidazolium chloride.

Figure 6 shows the variation of water flux as a function of duration of filtration at various applied electric field frequencies. Since pre-compaction has already been carried out for all the membranes, a stable water flux was noticed for almost all membranes with time. For base membrane, the application of external oscillating electric field did not show any effect on the flux values and variation. Even after application of 1kHz, electric field frequency, no change was observed. This is expected as the base membrane is not electrically activated. The electrical responsive membranes showed different behavior in the presence and absence of an externally applied oscillating electric field. For most of the responsive membranes, the trend was found to be similar. By the application of an electric field, of frequency 20 Hz, the water flux values were found to reduce considerably. The presence of ionic imidazolium moieties in repetition in PIL brush make the polymer electrically activated. In presence of applied oscillating electric field, the polymer brush tries to orient themselves depending on the polarity. This movement of polymer brush generates local perturbation, which is proposed to be sufficient for breaking of concentration polarization near the boundary layer and significantly improves the antifouling characteristics of the membranes. There was a statistically significant reduction in flux with increase in electric field frequency. However, the displacement in poly (ionic liquid) chains due to the interaction of the electric field generated by these brushes and externally applied oscillating electric field is inverse to the frequency of the oscillating electric field frequency [15].

(3) $$d=\;\frac{F}{m}\;\left(\frac{1}{f^{2}}\right)$$

where *d* is the lateral displacement due to local perturbation, *F* is force of magnetic field, and *m* is the effective mass. This implies that the local perturbation is more in 20 Hz compared to the 1-kHz oscillating electric field. More the local perturbation, more is the obstacle and lesser will be the flux. In view of this, the flux for 1 kHz should be more than that of 20 Hz. At higher frequency, the brushes would not get enough time for their movement. However, this would lead to the generation of localized heating. Moreover, the lesser heating could be attributed to the permeability, and hence the flux. This effect would have a more negative impact on the 1-kHz field compared to 20 Hz. Hence, both the factors of local perturbation and localized heating have entirely opposite effects on flux value. The observed water permeability is the cumulative effects of these factors. The resulting power due to the electrical interaction is given by [18]

(4) $$P=\;\pi \mu_{0}\chi_{0}H^{2}f\;\frac{2\pi f\tau }{1+{\left(2\pi f\tau \right)}^{2}}$$

where, *P* is the generated power, *f* is the frequency, *χ*_0_ is the electrical susceptibility, *μ*_0_ is the permeability, and *H* is the amplitude of the electric field. Briefly, the more power, the greater the localized temperature. 

**Figure 7:**
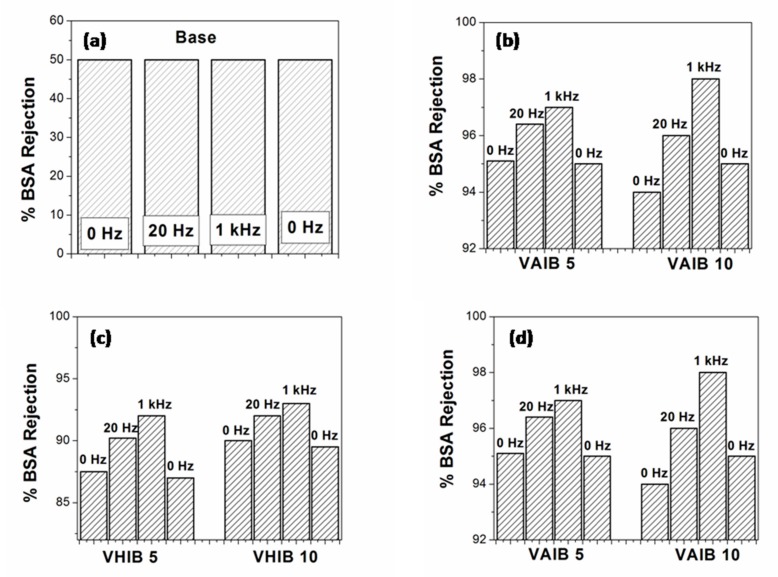
The rejection of BSA at various oscillating electric field frequency for the responsive membranes. (**a**): Virgin RCUF membrane; (**b**): Responsive membranes originated from vinyl allyl imidazolium bromide; (**c**): Responsive membranes originated from vinyl hexyl imidazolium bromide; (**d**): Responsive membranes originated from vinyl butyl imidazolium chloride.

Figure 7 shows the % rejection for BSA in pH 7. The base RC UF membrane showed ~50% BSA rejection and it remained unchanged even after the application of oscillating electric fields of 20 Hz and 1 kHz frequencies. However, electric responsive membranes showed different responses in the presence of applied oscillating electric field of different frequency. At lower frequency (20 Hz), the movement of PIL brush due to electric field interaction provides additional barrier for the BSA molecules to pass through the membrane and hence the enhancement in % rejection was observed. The presence of an oscillating electric field of higher frequency (1 kHz) led to the enhancement of the % of BSA rejection. However, the enhancement was only marginal. The localized heating might be responsible for the same. All the responsive membranes showed an almost similar trend. The responsive membranes obtained from longer duration of initiator immobilization, also showed marginally higher % rejection of the BSA. At pH 7, the BSA molecules are expected to have negative zeta potential, which can have attractive interaction with the PIL brush, which is mostly positively charged due to the presence of repetitive units of imidazolium ring. It is believed that the counter ions associated with each imidazolium ring is mobile in nature, especially in the presence of aqueous medium. Therefore, the enhancement in % rejection can be counterbalanced by the electrostatic interaction. The BSA flux was also monitored as a function of filtration time and was found to follow similar trend as seen in case of water flux.

The size exclusion chromatographic analysis using dextran of different molecular weight have been utilized to understand the molecular weight cutoff of these modified membranes and the effect of oscillating electric field on the molecular weight cutoff [30]. The rejection curves for all the responsive membranes are shown in Supplementary Figure S1. Table 3 summarizes the molecular weight cut off for the electric responsive membranes in presence of oscillating electric field of different frequency. The modified membranes were found to have reduced molecular weight cutoff compared to the virgin membrane indicating partial pore blockage or extensive growth of PIL brush on pore mouth. The responsive membranes with higher PIL density were found to have further reduced molecular weight cutoff. Application of oscillating electric field influenced the MWCO (molecular weight cut off) as it is based on the % of dextran rejection. At 20 Hz, the PIL brush will interact with the electric field resulting local perturbation, which would enhance the % rejection of the dextran molecules of higher molecular weight and consequently the MWCO was found to reduce. In the 1 kHz electric field, the MWCO was further reduced. However, the extent of reduction was not very high. The local perturbation or the movement of PIL brush did not induce any irreversible change on membrane, as evidenced by the similar MWCO at 0Hz, even after the application of a 1-kHz oscillating electric field.

## 4. Conclusions

Electric responsive UF membranes were prepared by the controlled growth of PIL brush on RC membranes to create local perturbation by the application of an external oscillating electric field in order to avoid concentration polarization. PIL grafting induces hydrophilicity on the membrane surface by incorporating an electrostatic interaction through charged imidazolium ions. PIL grafting also induces surface roughness and a reduction in MWCO of the virgin membranes. In the presence of an oscillating electric field of 20 Hz frequency, the movement of PIL brushes affects the water permeation through the membrane. However, a very high frequency (1 kHz) additionally induces the localized heating due to the strong electric field interaction. Such localized perturbation and heating were found to influence the overall rejection of protein molecules having either a surface charge like BSA or a neutral surface like dextran. The localized perturbation creates an enhancement in the overall % of protein rejection. However, these effects arising from the electric field interaction between the charged PIL surface and the applied oscillating electric field were found to be reversible, which provides the opportunity for the remote control of the membrane performance depending upon the applications.

## Figures and Tables

**Table 1:** Dextran molecular weights, suppliers, and concentration used in the sample solution.

Dextran Fraction	MW (kD)	Concentration (g/L)
T6	6	1.00
T40	40	0.74
T70	70	0.34
T500	500	0.27

**Table 2:** The EDX analysis for the % compositions of hetero atoms in responsive membranes.

Element	Base	VAIB 10	VHIB 10	VBIC 10
C	54.98	51.07	53.16	48.91
O	45.02	14.26	10.71	19.71
N		18.23	23.47	15.47
Br		16.44	12.66	−
Cl		−	−	15.91

**Table 3:** The molecular weight cutoff (in kDa) for responsive membranes in presence of oscillating electric field of different frequency.

Membranes	0 Hz	20 Hz	1 kHz	0 Hz
Base	304	304	302	300
VAIB 5	239	202	188	234
VAIB 10	233	199	186	237
VHIB 5	246	216	190	241
VHIB 10	222	187	171	216
VBIC 5	214	190	173	209
VBIC 10	216	200	184	214

## Supplementary Materials

The following are available online at https://www.mdpi.com/1660-4601/17/1/271/s1, Figure S1: The dextran rejection curves for responsive membranes without any applied electric field based on size exclusion chromatography.
