# Supplementary material for: Designing Electric Field Responsive Ultrafiltration Membranes by Controlled Grafting of Poly (Ionic Liquid) Brush
Source: Int J Environ Res Public Health. 2019 Dec 30;17(1):271. doi: 10.3390/ijerph17010271 (PMC6981848; doi:10.3390/ijerph17010271)
Supplement: Supplementary file 1 [file ijerph-17-00271-s001.pdf]

Supplementary Information

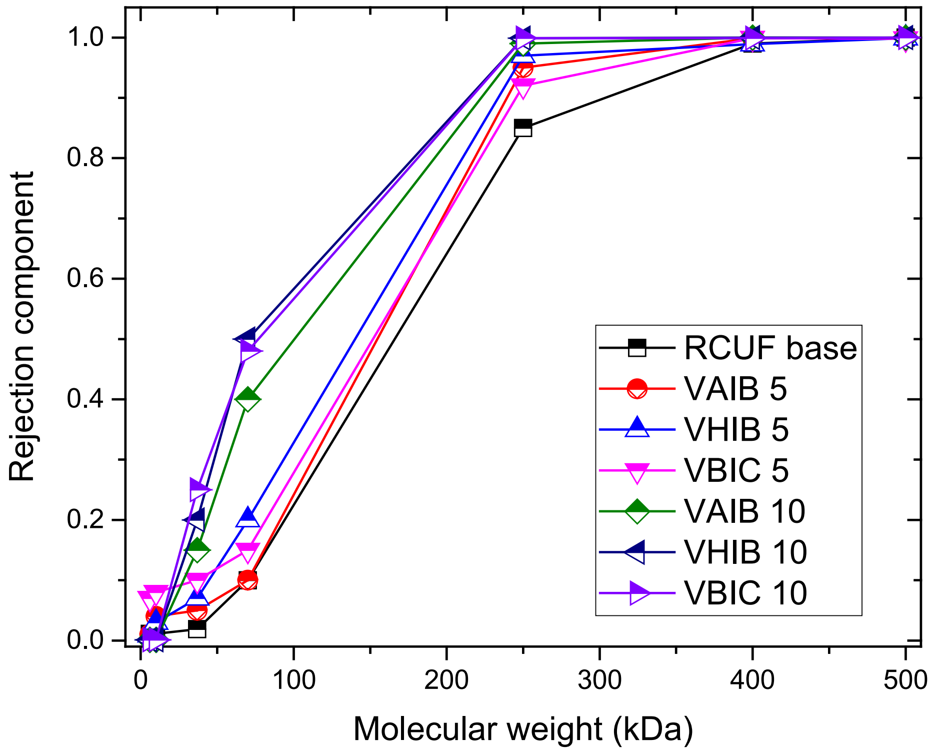

Figure S1. The dextran rejection curves for responsive membranes without any applied electric field based on size exclusion chromatography.
